# Exploring the Impact of Policies to Improve Geographic and Economic Access to Vegetables among Low-Income, Predominantly Latino Urban Residents: An Agent-Based Model

**DOI:** 10.3390/nu14030646

**Published:** 2022-02-03

**Authors:** Deborah Salvo, Pablo Lemoine, Kathryn M. Janda, Nalini Ranjit, Aida Nielsen, Alexandra van den Berg

**Affiliations:** 1 Prevention Research Center, Brown School, Washington University in Saint Louis, Saint Louis, MO 63130, USA; 2 Centro Nacional de Consultoría, Bogotá 110221, Colombia; plemoine@cnccol.com; 3 UTHealth School of Public Health, Austin, TX 78701, USA; Kathryn.M.Janda@uth.tmc.edu (K.M.J.); Nalini.Ranjit@uth.tmc.edu (N.R.); Aida.Nielsen@uth.tmc.edu (A.N.); Alexandra.E.VanDenBerg@uth.tmc.edu (A.v.d.B.)

**Keywords:** food policy, food environment, dietary behaviors, systems science, low-income groups

## Abstract

Modifying the food environment of cities is a promising strategy for improving dietary behaviors, but using traditional empirical methods to test the effectiveness of these strategies remains challenging. We developed an agent-based model to simulate the food environment of Austin, Texas, USA, and to test the impact of different food access policies on vegetable consumption among low-income, predominantly Latino residents. The model was developed and calibrated using empirical data from the FRESH-Austin Study, a natural experiment. We simulated five policy scenarios: (1) business as usual; (2)–(4) expanding geographic and/or economic healthy food access via the *Fresh for Less* program (i.e., through farm stands, mobile markets, and healthy corner stores); and (5) expanding economic access to vegetables in supermarkets and small grocers. The model predicted that increasing geographic and/or economic access to healthy corner stores will not meaningfully improve vegetable intake, whilst implementing high discounts (>85%) on the cost of vegetables, or jointly increasing geographic and economic access to mobile markets or farm stands, will increase vegetable intake among low-income groups. Implementing discounts at supermarkets and small grocers is also predicted to be an effective policy for increasing vegetable consumption. This work highlights the utility of agent-based modeling for informing food access policies.

## 1. Introduction

The multiple health inequalities affecting low-income minority populations in the United States (US) have been extensively described [1,2,3]. Low-income, minority urban residents have a significantly higher risk of developing diet-related outcomes, including obesity, CVD, type II diabetes, and certain types of cancer, than higher-income white populations [4,5,6,7,8,9]. Fruit and vegetable intake is a critical health behavior for preventing and controlling obesity and non-communicable diseases, given its role in preventing chronic inflammation, improving gut motility, increasing satiety, and preventing weight gain [10,11]. The US Dietary Guidelines recommend that adults and children consume five portions of fruits and vegetables per day, with vegetable intake recommended to be higher than that of fruit (2–3 cups per day versus 1.5–2 cups per day) [12]. However, a large proportion of residents of urban, low-income, predominantly minority communities continues to consume insufficient amounts of fruits and vegetables as part of their habitual diet, in detriment to their health [13,14]. While the prevalence of meeting the recommended levels of fruit intake is higher among Latinos than in the general population, vegetable consumption is a greater concern [15]. In the US, all racial/ethnic minority groups, including Latinos, as well as low-income residents, have lower levels of attainment of vegetable-specific intake recommendations (2–3 cups per day) than their white, high-income counterparts [16,17]. Increasing vegetable intake among low-income, predominantly Latino communities is therefore a public health priority.

Although both fruits and vegetables represent excellent sources of micronutrients and dietary fiber, there are some key differences between them. Because fruits constitute a natural source of sugar, which most humans are “programmed” to instinctively accept and enjoy, they tend to be more palatable and easily consumed by all [18,19,20,21]. Additionally, most fruits require shorter preparation time and equipment than vegetables, as they can be eaten raw, and are often consumed as snacks in between larger meals [22]. Vegetables, on the other hand, represent an acquired taste for many people [20,21], and some require more involved cleaning and preparation procedures for consumption [22]. The fact that we should ingest more vegetables than fruits per day, the low levels of vegetable intake among low-income minority communities, and the known challenges associated with the promotion of vegetable intake underscore the urgent need for identifying effective and sustainable strategies to increase vegetable intake among low-income, diverse urban populations in the US.

There are several examples of effective, inter-personal approaches for increasing fruit and vegetable intake among low-income minority groups, including specific ones designed for Latino populations [23,24]. However, there are also outstanding concerns. While these types of approaches yield statistically significant improvements in well-controlled trials, their effect sizes tend to be low to moderate, and evidence of maintaining sufficient vegetable intake over time is weak. Likewise, the reach and coverage of these types of approaches remain low. However, obesity has reached epidemic levels in the US, with 4 out of 10 adults suffering from obesity [25]. Although inter-personal strategies are helpful, on their own, they are unlikely to yield the population-level shifts in dietary patterns required to revert the crisis of obesity and associated chronic diseases in the US, which disproportionately affect low-income and minority groups.

Large-scale, systems-oriented solutions are required for reverting the obesity epidemic in the US. A large body of evidence supports a link between policy and environmental factors and healthy eating behaviors [26,27]. Specifically, geographic and economic access to food are known to be critical in shaping food consumption patterns in communities [26,27,28]. However, most studies examining the influence of the geographic and economic food environments on eating behaviors, and the effectiveness of large-scale strategies to modify the food environment in promoting healthy eating, are limited [29]. The majority of existing evidence comes from studies employing cross-sectional designs [30,31]. Additionally, among the few longitudinal studies available, the follow-up assessment timelines tend to be insufficient for observing meaningful changes in population food intake patterns, as they are restricted by traditional research funding and policy cycles [29].

Two under-utilized methods for assessing the impact of food policy and environmental changes on individual-level behavioral outcomes (e.g., vegetable consumption) are natural experiments and systems- and simulation-based approaches. The aim of this study was to develop, calibrate and use an agent-based model to simulate the impact of different types of food access policies on vegetable consumption levels among low-income, predominantly Latino urban communities in Central Texas. Our simulation exercise is unique in that it was directly informed by a natural experiment, which employed empirical methods and a longitudinal observational design to assess the impact of real-life, small-scale strategies aimed at increasing geographic and economic access to fresh fruits and vegetables through non-traditional food retail stores (farm stands, mobile markets, and healthy corner stores) [32]. Through our modeled scenarios, we examine the possibility of scaling up these types of strategies in varying ranges. Further, we contrast the potential impact of geographic versus economic access policies for improving vegetable intake among low-income, predominantly Latino communities and, of policies focused on improving access to non-traditional (farm stands, mobile markets, and healthy corner stores) versus traditional food stores (supermarkets and small grocery stores).

## 2. Materials and Methods

### 2.1. Study Context: Food Insecurity in Central Texas

Central Texas has experienced tremendous economic and population growth over the last decade [33]. However, this has been accompanied by salient disparities in access to healthy food by race/ethnicity and income in the region [34,35]. Specifically, Eastern Travis County, in the Austin Metropolitan Area, has historically had a lower median household income, fewer food retail outlets that sell healthy products, higher prevalence of food insecurity, and a higher proportion of Hispanic and Black residents than Western Travis County [34,35,36,37]. In response to these known spatial, ethnic/racial, and economic disparities in access to healthy food, the City of Austin developed the *Fresh for Less* program, a multi-level healthy food access initiative informed by formative research [38]. *Fresh for Less* aims to increase access to healthy food among low-income, diverse residents, by strategically placing non-traditional retail outlets, including farm stands, mobile markets, and healthy corner stores, in low-income areas of Eastern Travis County [38]. Farm stands refer to small pop-up markets that sell locally grown, fresh produce at subsidized prices [32,38]. Meanwhile, mobile markets are small pop-up markets that sell locally grown, fresh produce as well as healthy staple goods (eggs, canned vegetables, etc.) at subsidized prices [32,38]. Finally, healthy corner stores are participating convenience stores that agreed to carry a suite of healthy products including fresh produce, whole grains, low-fat dairy, low-sodium canned vegetables, and other products, informed by the Food Trust Healthy Corner Store model in Philadelphia [32,38,39].

### 2.2. The FRESH-Austin Study (Parent Study)

The agent-based model presented in this paper is one of multiple components of the Food Retail: Evaluating Strategies for a Healthy Austin (FRESH-Austin) Study [32]. The FRESH-Austin Study aims to comprehensively assess the City of Austin’s Fresh for Less Program and to increase our understanding of the complexities involved in designing and implementing effective food access policies in low-income, diverse urban communities [32]. As a whole, the study includes a robust primary data component set within the context of a natural experiment (constituting the expansion of the Fresh for Less program in Eastern Travis County). Data collection for this portion of the work took place from 2018 to 2021. The methods and baseline results of the empirical component of the FRESH-Austin study have been extensively described elsewhere [32].

Briefly, a cohort of 400 participants was used, which included a sample of participants who shopped at *Fresh for Less* assets, participants who lived within 1.5 miles of a *Fresh for Less* asset and thus were considered geographically exposed to the natural experiment, and participants who resided in areas that did not have any *Fresh for Less* assets but were similar, in terms of sociodemographic and urban design characteristics, to neighborhoods served by the *Fresh for Less* program (comparison group) [32]. The three-year cohort study included annual surveys and the use of wearable devices (accelerometers and GPS monitors) to objectively assess the spatial patterns of food store visits among a subsample of participants (*n* = 100). Qualitative data collection also took place among a sub-sample of participants who were part of focus groups. In addition to participant-level data, the FRESH-Austin study collected secondary GIS data pertaining to the food environment and conducted a series of micro-scale environmental audits to assess the built environment surrounding *Fresh for Less* locations. Food inventories were also performed at *Fresh for Less* assets.

The Baseline survey, GIS (store location) and inventory (food price) data were used to inform the decision-making process of agents and to calibrate the agent-based model presented in this paper [32]. For context, the majority of the sample identified as Latino (54.41%) reported earning under USD 45,000 in household income in 2017 (52.62%), and had a higher prevalence of food insecurity than the rest of the county (FRESH: 39.60%; Travis County: 12.90%). In terms of food purchasing and consumption behaviors, virtually all FRESH participants reported primarily shopping at supermarkets/large grocery stores (99.25%). Among participants, 50.25% ate less than 2 cups of vegetables per day. Table 1 presents the basic sociodemographic and food-related behavior characteristics of the FRESH-Austin Study cohort sample.

### 2.3. Agent-Based Model to Assess the Impact of Food Environment Policies on Vegetable Intake in Low-Income, Diverse Communities

#### 2.3.1. Agent-Based Modeling: An Under-Utilized Tool for Informing Public Health Policy

Agent-based modeling is a type of systems-based, simulation method, via which an environment, often based on a real-world setting (e.g., a city), its assets (e.g., locations of food stores), and residents (referred to as “agents”) are simulated [40,41,42]. Further, the way in which agents interact with their environment and its assets to ultimately make decisions is also simulated. Agent-based models are part of the family of complex systems or complexity sciences [40,41,42], and differ substantially from statistical models, in that the model does not constitute a single equation. Rather, the model is a system in itself, with multiple inter-dependent components, including: the modeled environment (e.g., a city), its specific environmental assets of interest for the research in place (e.g., the food environment), its simulated inhabitants (agents), and the rules that determine how agents interact with their environment to ultimately make decisions (e.g., food purchasing and intake decisions) [40,41,42]. When model parameters and decision rules are evidence based, and accordingly calibrated, agent-based models are helpful tools for testing “what if?” policy expansion scenarios. For example, what if a car-dependent city were to suddenly implement universal coverage of protected bicycle lanes and sidewalks? How would this major built environment modification impact active travel and overall physical activity levels among the population? Would there be unintended consequences [43]? The impact of large-scale, urban transformational strategies such as the one in this example is very difficult to assess using traditional empirical methods [43,44]. Given the lack of evidence on their real-world effectiveness, these types of policies are often considered too risky for decision makers to support, both in terms of their economic and political costs [44]. Agent-based modeling provides a powerful alternative for generating evidence on the possible impacts of large-scale policy expansion scenarios on population-level changes of health-related behaviors.

#### 2.3.2. Model Development

We developed a time-discrete, agent-based model to simulate the decision-making process leading to daily vegetable intake among adults (main outcome). The model was developed using Wolfram Mathematica, version 11.3. The multiple components of the agent-based model (simulated environment, food environment characteristics, agents, decision-making process, and policy expansion scenarios) are described in Section 2.3.2, Section 2.3.3, Section 2.3.4, Section 2.3.5, Section 2.3.6, Section 2.3.7 and Section 2.3.8, below.

#### 2.3.3. Modeled Environment

The model was built to simulate the food environment and adult population of the City of Austin, Texas, USA. The City of Austin’s metropolitan area has a total population of 1.8 million inhabitants, and an area coverage of 790 Km^2^. Austin is the political and economic capital of the state of Texas, and had a Gross Domestic Product per capita of USD 63,839 USD in 2017 [45]. Its racial/ethnic composition is 48.3% White, 33.9% Latino or Hispanic, and 7.8% Black [33]. The model is based on a raster map of the City of Austin of 6600 by 5100 pixels, stratified by income quantiles (modeled after 2017 American Community Survey data, by census tract). Hence, our model simulates the spatial distribution of income across the City of Austin (see Section 2.3.4, below), which serves as the base layer on top of which food vending assets (food stores and restaurants) and inhabitants (“gents”) were further simulated (see Section 2.3.5 and Section 2.3.6, below).

#### 2.3.4. Food Stores and Restaurants (Food Environment)

Elements of the food environment regarding places where people shop for food to consume at home were built in the modeled environment using objectively collected data by City of Austin’s Office of Sustainability, which had previously conducted a comprehensive inventory of all food vending locations in the city [34]. These data were shared with the FRESH-Austin Study research team in tabular format and included street address information for all supermarkets (*n* = 73), small grocers (*n* = 1161), convenience stores (*n* = 91), gas stations (*n* = 411), pharmacies (*n* = 58), and discount stores (43). All food vending locations were geocoded using ArcGIS version 10.6 (ESRI, Redlands, NC, USA), and integrated into the modeled environment. Because information on the location of restaurants was not included in the City of Austin’s inventory, we randomly placed these throughout the modeled environment, representing the full extent of the City of Austin. These included full-service restaurants (*n* = 100), casual restaurants (*n* = 300), and fast-food restaurants (*n* = 300). Meanwhile, non-traditional food vending locations, including mobile markets (*n* = 7), farm stands (*n* = 7), and healthy corner stores (*n* = 5) (i.e., the type of assets that the *Fresh for Less* initiative aims to increase access to), were randomly placed in low-income neighborhoods of the modeled environment. The number of non-traditional food assets included in the modeled environment for the business-as-usual scenario was consistent with the coverage of the *Fresh for Less* program in 2018 (baseline assessment year for the empirical portion of the FRESH-Austin Study).

The model assumes that food vending assets can sell three major food categories: vegetables, unhealthy foods (high in fat, sugar or salt), or other foods (grains and cereals, legumes, etc.). The use of three food categories was selected to ensure parsimony in the model (i.e., to keep the model as simple as possible, so long as the calibration showed satisfactory/plausible results; see Section 2.3.7 and Section 3.1 for calibration methods and results) [46]. One of the categories represents the outcome of interest (vegetables), while the other two represent all other foods, divided into those known to be unhealthy if consumed in excess (e.g., sugar-sweetened beverages, ultra-processed foods high in fat, sugar, and/or salt, etc.) [47,48,49], and all other foods (e.g., natural grains, cereals, legumes, etc.). The types of foods sold in each store, their price, perceived quality, and the estimated time it takes to consume them, as well as the variety of food items and services offered at each store type, were factors that were found to matter for store selection among participants of the FRESH-Austin Study, per survey-based (see Table 1) and additional qualitative data. In our model, the values and weights assigned to each of these factors were informed by a combination of empirical data collected at baseline via the FRESH-Austin Study (survey-based data, store audit/inventory data, and qualitative data from focus groups) [32,50], and input of the research team, which included nutrition, behavioral science, and epidemiology experts, with extensive knowledge of the food environment in the City of Austin. Specifically, information on the types of foods sold in each store type was derived from objective store audit/inventory data, as were the prices (USD per portion) of the three main categories of food included in our model (vegetables, unhealthy foods, other foods). The perceived quality of foods and perceived variety of products services offered at each store type were informed by qualitative data of this and prior studies in the same study area/population [32,50,51], and by expert opinion of the research team. It is important to mention that these “perceived” characteristics refer to the perception by the community that these stores serve. Perceived quality and store variety were each assigned a score ranging from 0 (lowest perceived quality/variety) to 10 (highest perceived quality/variety). The process for assigning each food type, within each store type, a perceived quality and variety store, consisted of (a) each research team member individually proposing a score, based on their knowledge of the community perceptions of the local food environment; (b) the team coming together to review everyone’s scores; (c) deciding on a final score for quality and variety per store type. When significant discrepancies in assigned scores across team members were observed, the team engaged in an open discussion to justify their selected scores, drawing from their past research findings from similar studies among the same population and/or in the same geographical region, FRESH-Austin Study findings from the qualitative data, and personal knowledge of Austin’s food environment and community values. These discussions continued until the team reached a consensus for all assigned scores. Finally, the time it takes to prepare and consume a meal including each of the food types, in minutes, was also informed by expert input, following an equivalent process for perceived quality and variety.

#### 2.3.5. Agents

The modeled environment was populated by 2100 agents, representing adult residents of the City of Austin. Six hundred agents were randomly assigned to reside in high-income neighborhoods, 900 were assigned to middle-income neighborhoods, and 600 were assigned to low-income neighborhoods, where the personal income level was assumed to match their residential income group (i.e., agents residing in high-income areas were assumed to have high income themselves, and so on). Each agent was assigned a weekly budget for food, corresponding to USD 210 for high-income agents, USD 161 for middle-income agents, and USD 105 for low-income agents. These quantities were informed by FRESH-Austin baseline cohort survey data. All agents were also assigned a given amount of time per day for food purchasing and consumption activities, including travel time to food stores and restaurants, cooking time for home-made meals, and actual eating time. Half of the agents across all income levels were assigned five hours per day for food purchasing and consumption-related activities, while the other half were assigned ten hours per day. The distinction in agents having more or less time available for food-related activities was meant to simulate agents that work (and have less time for food-related activities) and those that do not work (and have more time for these activities). Finally, all agents were assigned a given preference towards vegetable consumption, with half of the agents being three times more likely to buy vegetables as opposed to other food groups available to them at the same price.

#### 2.3.6. Agents’ Decision-Making Process

**Figure 1:**
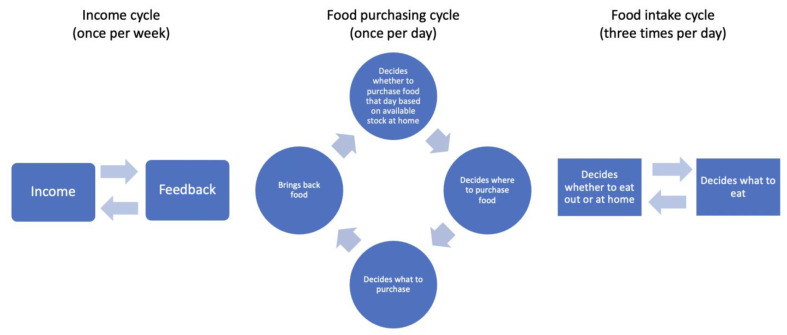
Three cycles of agents’ decision-making process for food purchasing and consumption.

The model assumed that agents must have three meals each day, as this is the most common meal frequency in the US, usually corresponding to breakfast, dinner and lunch [52]. Further, each meal was assumed to be composed of three portions of food, as this is a common way in which dietary guidelines describe the composition of meals (e.g., US dietary guidelines specify that one should consume a portion of fruits and/or vegetables, protein, and grains in each meal [53]). Our model assumed that each portion of food could consist of one of the three food categories simulated: vegetables, unhealthy foods (high in fat, sugar, and/or salt), and other foods (grains and cereals, legumes, etc.). The decision-making process leading to food intake in the model is composed of three inter-related cycles, which yield each agent’s decisions on which foods to consume for their 21 meals per week (3 meals per day). Figure 1 shows the overall structure of the model based on the three cycles, which operate at the weekly, daily, and meal-based epoch levels.

The first cycle occurs on a weekly basis and relates to the weekly food budget available to each agent per their income category (high, medium, or low). The model assumes that agents cannot spend more money than what they have available for food each week (i.e., the model does not account for real-life financial tools such as credit). The decision to use a weekly cycle for food spending was based on the fact that the majority of the sample reported shopping for food at least once per week (see Table 1).

The second cycle occurs on a daily basis because humans need to eat every day, and because the real-life residents of Austin have the option of buying food on any given day of the week (open stores and restaurants are available on a daily basis). As such, the model assumes that every day, before the first meal of the day, each agent goes through a process of verifying how much food they have available at home. If on any given day an agent has less than five portions of food remain at home (i.e., less food than needed for two full meals), they will be a assigned a probability of 1 for shopping for more food that day, whilst if more than five portions of food are available at an agent’s home, the probability of shopping for food that day decreases, per a logistic function. Next, agents that end up shopping for food on a given day must decide where they will do their shopping. This decision is based on the food vending locations the agent can afford, based on a function of distance, cost of food, consumption time of foods sold, variety of items and services available, and the quality of the food sold at each store. Hence, this decision step relies on the available time, food budget, and assigned preferences for each agent. Every time an agent decides they will shop for food, select a store, and determine how many portions of food of different types they will purchase, the given amount of money spent on their purchase is deducted from their available weekly budget for food.

The third cycle occurs at every meal, with meals occurring three times per day, the most common meal frequency in the US (breakfast, lunch and dinner) [52], and with each meal consisting of three portions of food [53]. Agents first decide if they want to eat at home or if they will eat at a food service location (restaurant). The same function used in the daily cycle (cycle 2) for selecting where to shop for food was applied, but now with restaurants included as the available locations. If an agent decides to eat a meal at home, they select their three meal portions based on which foods they have in stock, their food preferences, and the time they have to prepare and eat their food. Every time an agent eats a given portion of food at home, it is deducted from their available food stock, whilst each time an agent eats out at a restaurant, the money spent on the given meal is subtracted from their available weekly budget for food.

Once all cycles are over for the seven days composing a week, including 21 meals (3 per day) and 63 portions (3 per meal), the model implements a change in the amount of portions required for each given agent. This change is proportional to the portions of unhealthy foods (high in fat, sugar, and/or salt) that the agent consumed during the given week, minus the number of vegetable-based portions consumed during the same week. This *feedback loop* is implemented to simulate the fact that diets high in fat, sugar and salt appear to increase appetite and lead to progressive overeating, a phenomenon that has been described as the *Salted or Unhealthy Food Addiction Hypothesis* [47,48].

#### 2.3.7. Model Assessment

We assessed the model through two calibration steps. First, the decision of where agents shop was calibrated to fit the observed number of visits per month to supermarkets, since our data from the empirical portion of the study showed that under real-life circumstances at baseline (2018), most visits to food stores (99.25%) are to supermarkets, and this is true across all socioeconomic strata [32]. This baseline situation constitutes the “business-as-usual” scenario in our model. With the calibration, we compared the empirical baseline data from the FRESH cohort study to the modeled results of visits to a supermarket per month, stratified by three levels of money available for food (to simulate the high-, medium- and low-income groups).

The probability of choosing store i was estimated with the following function: P(Storei) ∝ P(Cost of food)+P(distance)+P(time of consumption)+3* P(variety)+4* P(quality).

where:

$$\begin{array}{ccc} P\left(Cost\;of\;food\right) & = & \frac{\;Agents\;available\;money\;for\;food-Average\;cost\;of\;the\;store}{\sum^{\;}Agents\;available\;money\;for\;food-Average\;cost\;of\;all\;stores}\\ P\left(distance\right) & = & \frac{\;1/distance}{\sum \begin{array}{c} \frac{1}{distance}\;For\;all\;stores \end{array}}\\ P\left(Consumption\;Time\right) & = & \frac{\;Agents\;available\;time-average\;time\;of\;consumption}{\sum^{\;}Agents\;available\;time-average\;time\;of\;consumption\;for\;all\;stores}\\ P\left(variety\right) & = & \frac{\;Variety^{3}}{\sum^{\;}\;Variety^{3}\;For\;all\;stores}\\ P\left(quality\right) & = & \frac{Quality^{3}}{\sum^{\;}\;Quality^{3}\;For\;all\;stores} \end{array}$$

* For all formulas above, only values from 0 to 1 can be produced. If a negative value is obtained, it is capped at 0. Values above 1 are not mathematically possible.

The elements included in the formula above are meant to represent the influence that each factor can play in the probability of choosing a store based on it (i.e., the probability of choosing a store based on (a) its cost, (b) its distance from home, (c) the time it takes to consume the foods sold in that store, (d) the variety of items and services available at the store, and (e) the quality of the items sold). These factors, and their weights in the formula, were selected given their salience for food purchasing decisions, in accordance with the mixed-methods findings from the empirical baseline data of the FRESH-Austin evaluation and expert input from the authors of this paper.

The second assessment step that we conducted was a calibration of the agents’ behavior in response to the cost of food. Previous studies have shown that people of lower income tend to consume fewer vegetables than higher-income populations but are more sensitive to changing their behavior in response to changes in food prices (i.e., have higher price elasticity) [54,55,56]. Our model simulates this behavior. Under a scenario of 0% discount on the cost of vegetables, the lower-income group consumes fewer portions of vegetables per meal than the middle- and high-income groups. However, as the relative (%) discount on the cost of vegetables increases (i.e., as vegetables become cheaper), the low-income group increases the portions of vegetables that they consume per meal, and this occurs at a higher rate than in the other income groups, since the model was built to simulate the increased sensitivity to price changes (elasticity) among the lower-income group.

#### 2.3.8. Policy Scenarios and Outcome of Interest

We simulated five scenarios, including a business-as-usual scenario, three *Fresh for Less* policy expansion scenarios, and one scenario that harnesses cost incentives in traditional food vending outlets (supermarkets and small grocers), as a means to increase vegetable intake among low-income groups (main outcome).

##### Business-as-Usual Scenarios

This scenario represents the real food environment in Austin, Texas, as it was at baseline (2018) for the FRESH-Austin Study and serves as the comparison scenario against which all other simulated policy expansion scenarios were contrasted to. This is the scenario for which model assessment (calibration) took place.

##### Fresh for Less Policy Expansion Scenarios

We modeled three scenarios simulating improvements in access to vegetables among low-income, urban populations, through Fresh for Less initiatives, which rely on non-traditional food vending outlets such as mobile markets, farm stands, and healthy corner stores.

The first policy expansion scenario focuses exclusively on improving the geographic access to these non-traditional food vending outlets. Under this scenario, the density of Fresh for Less assets in low-income areas was increased, by simulating the placement of new mobile markets, farm stands, and healthy corner stores in low-income neighborhoods. For this scenario, 18 independent simulations were run per added store, giving a total of 1620 simulations (18 simulations for each new store added, with a range of 0 to 30 stores examined, for a total of 540 simulations per store type [18 × 30], with three types of stores included: mobile markets, farm stands, and healthy corner stores).

The second scenario focused on improving economic access to Fresh for Less stores by simulating varying levels of discounts in the cost of vegetables offered only in the existing locations of non-traditional food vending outlets in low-income neighborhoods (the number and location of existing locations were per the business-as-usual scenario). For this scenario, a total of 900 simulations were run (300 per store type), with 30 simulations for each 10% decrease in the cost of vegetables, with a range of 0 to 100% discount on the cost of vegetables examined.

Finally, the third Fresh for Less policy expansion scenario combined improvements in geographic and economic access to vegetables via non-traditional food outlets. With this scenario, we simulated the placement of new mobile markets, farm stands, and healthy corner stores in low-income neighborhoods, all of which offered a fixed 50% discount on the cost of vegetables, as modeled by the *Double Up Food Bucks* model [57]. For this scenario, 900 simulations were run (300 per store type), with 30 simulations per added store, with a range of 0 to 30 stores examined.

##### Reduced Cost of Vegetables in Supermarkets and Small Grocers Scenarios

One final scenario modeled focused on improving economic access to vegetables by focusing on the most predominant type of stores that people use for buying food (per our empirical FRESH-Austin study data at baseline): supermarkets and small grocers. Under this scenario, we simulated the use of discounts in the cost of vegetables at these traditional food vending outlets as a means to improve access to vegetables for all. Importantly, this scenario examines the impact of the cost-reduction strategy for all income groups in Austin, Texas, since it does so through the existing locations of supermarkets and small grocers in the city, which, unlike the *Fresh for Less* food vending outlets, is not restricted to low-income neighborhoods. For both grocery stores, and supermarkets, a total of 900 simulations were run (300 per income tertile), with 30 simulations per 10% decrease step in the cost of vegetables, per income tertile.

## 3. Results

### 3.1. Model Calibration Results

**Figure 2:**
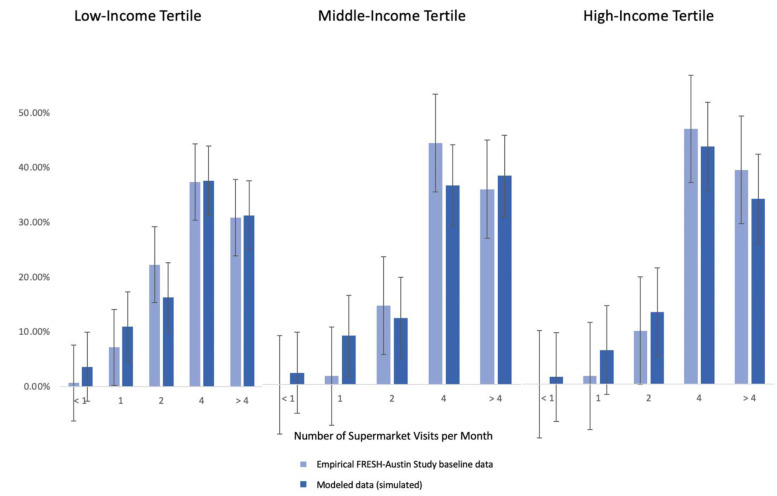
Agent-based model calibration based on frequency of visits to supermarkets.

**Figure 3:**
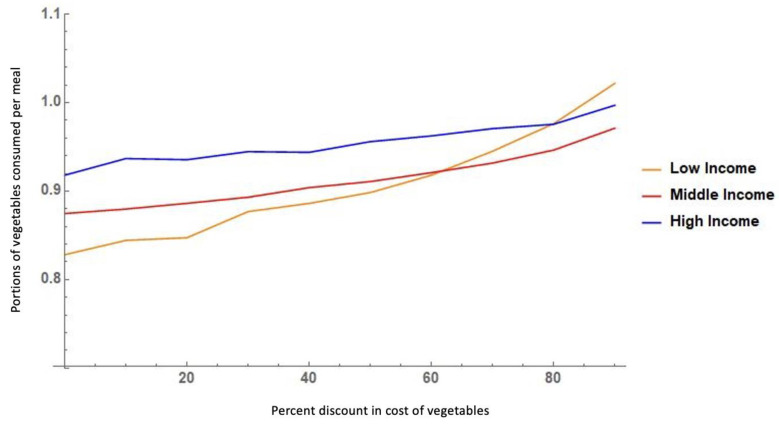
Agent-based model simulated response to discounts in the cost of vegetables by income level.

The agent-based model of the business-as-usual (baseline) scenario was found to adequately reflect the patterns observed in our baseline empirical data of the FRESH-Austin Study, as shown by the results of our two model assessment steps. This included calibrating the model against observed data on the frequency of visits to traditional food vending stores (supermarkets) (Figure 2), and on the price elasticity of vegetables among low-, medium- and high-income residents (Figure 3). Figure 2 shows that there was no statistically significant differences in terms of the distribution of visit frequency to supermarkets between our modeled versus real data. Likewise, Figure 3 shows that our model adequately reflects the fact that vegetable purchasing and intake behaviors among low-income residents are more sensitive to changes in the cost of vegetables.

### 3.2. Fresh for Less Policy Scenarios

**Figure 4:**
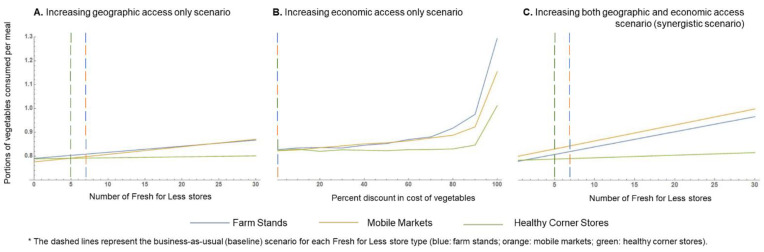
Agent-based model simulation results for *Fresh for Less* policy expansion scenarios.

Figure 4 shows the results of the simulations examining the predicted impact of different strategies to increase access to vegetables among low-income residents in Austin through the Fresh for Less program, employing farm stands, mobile markets, and healthy corner stores as food vending assets.

Relative to the business-as-usual scenario (baseline), expanding geographic access from 7 to 30 available mobile markets in low-income areas was expected to yield an extra 0.06 portions of vegetables consumed per meal. This would account to 0.18 added portions of vegetables per day among low-income community members. An equivalent increase in vegetable intake among low-income residents was predicted in the “economic access only” expansion scenario, if all existing mobile markets or farm stands were to offer a 70% discount for vegetables. Under the same policy expansion scenario, it was predicted that if the reduction in the cost of vegetables were to become greater than 85% in any *Fresh for Less* store type, the gains in extra portions of vegetables consumed per meal would become more meaningful, with greater expected gains occurring due to purchases at farm stands (about 0.15 added portions per meal (0.45 per day) at a 85% discount, and 0.47 added portions per meal (1.41 per day) if vegetables were offered free of cost). Finally, synergistically addressing geographic and economic access could result in a gain of up to 0.16 added portions of vegetables consumed per meal among low-income residents, if 23 new mobile markets were to be placed in low-income neighborhoods, whilst offering a 50% discount on the cost of vegetables. This would account for an extra 0.48 portions of vegetables consumed per day among low-income residents compared to business as usual. Under the same scenario, doubling the number of existing mobile markets at baseline (i.e., going from 7 to 14 mobile markets in low-income neighborhoods) is predicted to result in an extra 0.08 portions of vegetables consumed per meal (an added 0.24 portions of vegetables per day). For all modeled policy expansion scenarios, gains in portions of vegetables consumed per meal due to improved access to healthy corner stores (geographic, economic, or a combination of both) were either minimal or null.

### 3.3. Improving Economic Access to Vegetables via Traditional Food Stores Policy Scenario

**Figure 5:**
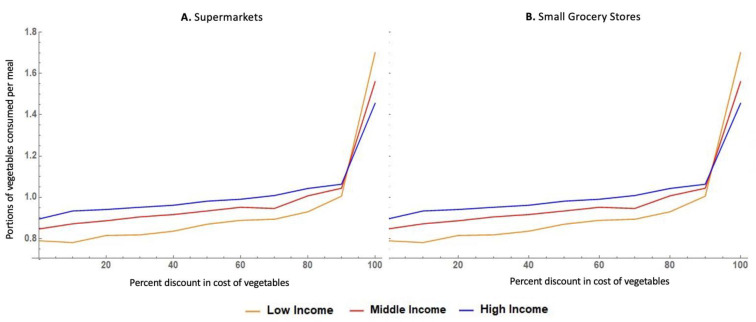
Agent-based model simulation results for expanded economic access to vegetables via small grocers and supermarkets scenario.

Figure 5 shows the results of the simulations examining the predicted impact of reducing the cost of vegetables at supermarkets and small grocery stores.

The pattern observed for the relation between the price reduction and the expected number of portions of vegetables consumed per meal was virtually the same across small grocery stores and supermarkets. A 50% discount on the cost of vegetables in either small grocery stores or supermarkets was predicted to yield a 0.06 increase in the portions of vegetables consumed per meal among low-income residents, i.e., 0.18 extra portions of vegetables per day. Reducing the price of vegetables by 90% of its original cost or more was predicted to yield substantially higher increases in vegetable consumption for all income groups. With a 90% discount on the cost of vegetables, an extra 0.21 portions of vegetables were predicted to be consumed per meal among low-income residents, equivalent to 0.63 added portions of vegetables per day. If vegetables were offered free of cost to low-income residents in supermarkets or small grocery stores, our modeling results predict an added 0.91 portions of vegetables to be consumed with every meal among low-income residents (i.e., 2.73 added portions per day).

## 4. Discussion

Through this study, we examined how modifications to the food environment of cities, specifically through policies, aimed to increase geographic and/or economic access to healthy food impact vegetable consumption among low-income urban residents. We achieved this by developing, calibrating, and implementing an agent-based model simulating the food environment of Austin, Texas, USA, and its influence on vegetable purchasing and consumption patterns. The model was informed by primary data collected as part of the FRESH-Austin Study [32], a natural experiment assessing the impact of the *Fresh for Less* initiative, which aims to improve access to fresh fruits and vegetables among low-income, diverse communities in Central Texas, via non-traditional food vending outlets (farm stands, mobile markets and healthy corner stores). Among the policy expansion scenarios explored, healthy corner stores were predicted to be the least effective in achieving higher vegetable consumption among low-income residents in Austin. Meanwhile, offering free vegetables at all traditional stores, where most residents currently shop for food (supermarkets and small grocers), is expected to yield the highest benefits in terms of increased vegetable intake for the target population (low-income, predominantly Latino communities). Beyond the extremes of the “best” or “worst” strategy, the results of the different scenarios simulated show that similar meaningful gains in vegetable intake among high-need groups can be achieved via different policy avenues (and considering different levels of scale-up needed). As such, our findings provide important insights that can help inform policy design and expansion plans in Central Texas for improving vegetable intake in high-need groups, both within and beyond the current scope of the *Fresh for Less* program.

Although some studies have highlighted the promise of healthy corner stores as a potentially effective strategy to improve the access to and consumption of healthy foods among low-income populations [58,59,60], our modeling results suggest that investing in scaling up this strategy is unlikely to yield meaningful gains in vegetable consumption among low-income, urban residents. The predicted lack of effectiveness in improving vegetable consumption among low-income residents was consistent across the three *Fresh for Less* policy expansion scenarios modeled, which included focusing exclusively on increasing the number of participating healthy corner stores within low-income neighborhoods (improving geographic access), focusing only on offering discounted prices for buying vegetables at existing corner stores (improving economic access), or jointly expanding the geographic coverage whilst implementing a 50% cost reduction for vegetables. Notably, the City of Austin and participating partners have recently decided to de-scale this strategy, which is no longer part of the core initiatives of the *Fresh for Less* program. The City has recently been working on re-designing this strategy to make it more effective. Our simulation-based findings provide evidence supporting this decision from a cost–benefit perspective and are also consistent with recent reports on the effect of healthy corner store programs for improving food purchasing and intake behaviors in other settings. A randomized-controlled trial in low-income neighborhoods in Philadelphia, PA, USA, examined changes in total energy intake among low-income youth due to a healthy corner store intervention, and reported no significant effects [61]. Likewise, a qualitative study in rural North Carolina reported corner store owners perceiving that fresh produce is not in high demand among their customers [58].

On the other hand, our model suggests that, if implemented at a larger scale, mobile markets and/or farm stands could become effective strategies for improving vegetable consumption among low-income, diverse urban residents if implemented at scale. Improving geographic access alone (i.e., increasing the number of stores in low-income neighborhoods) was found to be, relatively speaking, the least effective strategy for improving vegetable intake among low-income residents. For instance, if the City of Austin were to focus on expanding geographic access to mobile markets, an extra 0.18 portions of vegetables consumed per day among low-income residents (equivalent to 6–9% of the total daily recommended vegetable intake) could be achieved; however, this would require scaling up the program by 429% relative to its baseline operations in Central Texas in 2018. On the other hand, investing exclusively in improving economic access to vegetables through already existing mobile markets or farm stands could yield important benefits, but the price-discount threshold required for meaningful change to occur in terms of vegetable intake may be too prohibitive for this to be a feasible and sustainable approach (discounts of >85% in the cost of vegetables). Not unexpectedly, perhaps the most attractive policy expansion option, when considering the yield in terms of added vegetable consumption among the target audience (low-income residents), its feasibility, and the cost of implementation, was the synergistic scenario, whereby more stores are placed in low-income neighborhoods, whilst offering a 50% discount on the cost of vegetables. Under this scenario, a 200% increase in the number of available stores would result in 0.24 added portions of vegetables consumed per day among low-income residents (equivalent to 8–12% of the recommended daily vegetable intake). This type of strategy could be progressively implemented and scaled up as more resources become available, and key partnerships agreements are achieved (e.g., subsidizing vegetables through public assistance programs) [62,63].

Beyond the *Fresh for Less* initiative, representing a real-life strategy by the City of Austin for addressing food security and improving access to healthy eating in low-income, ethnically diverse communities, we also used our model to explore the potential impact of other hypothetical policy expansion scenarios on vegetable intake. Our modeling results suggest that reducing the cost of vegetables in the stores mostly used by community members to buy foods (supermarkets and small grocery stores) is a promising approach for improving vegetable intake among low-income residents. Empirical evidence from other studies supports “healthy food discounts” in supermarkets as an effective strategy for increasing fruit and vegetable intake in communities [64]. However, most studies have implemented the discounts in conjunction with other strategies (e.g., nutrition education, extra space allocation for produce), as part of multi-pronged interventions [64,65]. This makes it difficult to determine which intervention components are responsible for improvements in healthy eating behaviors. Another study in Michigan that offered SNAP-eligible participants a matching subsidy up to USD 20 per day off of locally grown produce in participating grocery stores found that the discount resulted in greater fruit and vegetable purchasing among SNAP-eligible customers; however, fruit and vegetable consumption was not measured [66]. Likewise, a Danish study found that a price-reduction intervention, combined for increased space allocation in supermarkets, led to a significant increase in fruit and vegetable intake, which was primarily driven by higher vegetable consumption [65]. This is consistent with our modeling predictions.

Notably, our model predicted that a 50% discount on the cost of vegetables across all supermarkets or small grocery stores, made available to all residents in the lowest tertile of income, would be as effective a strategy for increasing vegetable intake among low-income residents as expanding the number of available mobile markets in Austin by 429%. Our results are consistent with those of Widener et al. [67], who also developed an agent-based model to test different strategies to increase the purchasing of fresh produce (fruits and vegetables) in Buffalo, NY, USA. They reported only a modest increase in fruit and vegetable purchases due to an increased number of mobile markets in the city, as well as in response to a similar strategy than the healthy corner store initiative in Austin. On the other hand, a 50% discount on vegetables at grocery stores and supermarkets for low-income residents could be a feasible strategy, as evidenced by the success of the *Double Up Food Buck* program in other states among SNAP-eligible customers [55]. Having access to this type of information, regarding the relative impact of different policy expansion scenarios to reach similar behavioral targets, has high relevance for local policy makers and stakeholders across the food system, as it allows one to contrast possible avenues (and their implementation cost and complexity) for improving access to healthy eating, beyond those currently being implemented [40].

Our results must be interpreted in light of the limitations of the study. First, the focus of the agent-based model was on simulating the decision-making process for vegetable intake. Hence, our findings cannot be extrapolated to fruit (or any other healthy food group) intake. Second, the model has several assumptions which must be considered when using its results to inform policy action. Like any agent-based model, the complex ways (including the number of variables involved) in which people make decisions are over-simplified by a set of standard rules and parameters in our model, which are applied to all agents. For instance, only the location of the home residence of agents was simulated, and agents are assumed to spend all of their time at or near their home. We accounted for employment status as an element that influences vegetable intake behaviors by assigning less time for buying, preparing, and eating food to some agents (representing those that are employed). However, this does not account for the fact that in real life, people travel to food stores from other locations beyond their home (e.g., one can stop to buy groceries after leaving work, and then head home). This type of spatially explicit interaction with broader locations in the city is not captured by our model. Third, our model is time invariant, and as such, does not account for the time it may take to observe changes in agents’ behavior due to policy expansions, or if effects change over time. Fourth, the weights assigned in the function used to predict the probability of selecting a given store to purchase food were partially informed by real data from the FRESH-Austin Study, from past studies in the same setting and population [51,68,69,70], and by expert opinion by the author team. While our model calibration suggests that these weights adequately reflect the value community members assign to different factors when selecting a food store, it is possible that in other settings or low-income populations the weight allocation would vary. Finally, our analysis presents the results of a finite number of simulations of select policy expansion scenarios. Many other policy expansion scenarios (or combinations of scenarios) could have been explored, but were beyond the scope of the current paper, and our computational resources. These could have included the effect of placing non-traditional food outlets, such as those included in the *Fresh for Less* initiative, in strategic locations (e.g., next to supermarkets), or exploring if jointly expanding geographic and/or economic access to multiple types of stores yields additive or multiplicative benefits in terms of vegetable intake gains.

Our study also had many strengths. This is the first ever agent-based model simulating the food environment of the City of Austin, its influence on individuals’ vegetable consumption and intake patterns, and the potential impacts of policies to increase access to healthy foods on vegetable intake among low-income, predominantly Latino populations. Our model assessment results showed that the business-as-usual scenario adequately reflects the interactions of people with their food environment (in particular, with respect to shopping frequency and the role that price reductions can have in vegetable intake for low-income populations). This supports the credibility of the results simulating hypothetical policy expansion scenarios, and the expected gains in terms of increased vegetable intake among low-income, predominantly Latino communities. Finally, all of the modeled scenarios have realistic implementation potential for Austin, Texas, either by scaling up specific components of the existing *Fresh for Less* program, and/or by expanding of the *Double Up Food Bucks* program, in order to improve geographic and economic access to healthy foods.

## 5. Conclusions

Our work highlights the utility of agent-based modeling for food access, food policy, and public health research at large [40,43], by providing important, action-oriented evidence that can help inform equity-based food access policy priorities in Central Texas, US. In particular, our modeling results suggest that food environment policies should not heavily rely on healthy corner stores as a strategy to increase vegetable intake among low-income, predominantly Latino communities in Austin. Meanwhile, offering vegetables 100% free of cost for low-income residents at all supermarkets or small grocers has the potential to yield notable gains in vegetable intake among these communities. However, such an approach could have considerable economic, political, and/or implementation challenges. On the other hand, our model provides slightly less effective, but perhaps more realistic alternatives for policy makers to consider (e.g., a synergistic approach increasing geographic and economic access to *Fresh for Less* assets, and/or a substantial increase in geographic access alone to *Fresh for Less* assets, and/or offering free vegetables only at *Fresh for Less* assets). Indeed, the ability to identify several policy avenues for action towards a common objective is one of the key benefits of agent-based modeling. With this type of information, stakeholders can make informed decisions on the best course of action in their local setting by carefully considering the costs, benefits and challenges associated with the implementation and scale up of each strategy. More public health nutrition studies are needed that simultaneously employ empirical data collection (preferably, employing longitudinal designs), and systems-based, simulation approaches, and in which both methods inform each other in a continuous, ongoing fashion (through feedback loops). This type of approach could help achieve the promise of an efficient food systems *research-to-policy-action* pipeline.

## Figures and Tables

**Table 1:** Sociodemographic and food-related behavioral characteristics of the FRESH-Austin Study cohort sample, at baseline (2018) [32].

Variable	Strata/Category	%(*n*)/Mean [sd]
Total		400
Gender	Female	70.50 (282)
	Male	29.25 (117)
Age		43.89 [13.66]
Race/Ethnicity	Hispanic/Latino	54.41 (216)
	Black	10.08 (40)
	White/Other	35.52 (141)
Yearly household Income	Under USD 25,000	23.04 (88)
	USD 25,001–USD 45,000	29.58 (113)
	USD 45,001–USD 65,000	18.32 (70)
	> USD 65,000	29.06 (111)
Educational attainment	<High school	12.12 (48)
	High school or GED	21.72 (86)
	Some college	21.21 (84)
	Full college or more	44.95 (178)
Food assistance	Food bank user	12.00 (48)
	Free or reduced lunch user	26.50 (106)
	SNAP user	17.50 (70)
	WIC user	9.25 (37)
Food insecurity	Sometimes or often	39.60 (158)
	Never	60.40 (241)
Food purchasing frequency	Less than once per week	14.79 (59)
	Once per week	42.36 (169)
	More than once per week	42.86 (171)
Shopping locations (non-mutually exclusive)	Supermarkets	99.25 (397)
	Small grocer	64.75 (259)
	Convenience store	22.25 (89)
	Farmer’s market	12.25 (49)
	Mobile market	15.25 (61)
	Farm stand	13.00 (52)
Most important factor when deciding where to shop for food	Quality of food	52.63 (210)
	Cost	25.96 (101)
	Variety of food	12.34 (48)
	Quality of store	4.88 (19)
	Cultural variety	2.83 (11)
Vegetable purchasing (pounds/capita/week)		4.65 [3.93]
Vegetable intake (cups/day)		2.01 [0.96]

## Data Availability

Data can be shared by request and contacting the authors.

## References

[1] Bailey Z.D., Krieger N., Agénor M., Graves J., Linos N., Bassett M.T. (2017). Structural racism and health inequities in the USA: Evidence and interventions. Lancet.

[2] Payne-Sturges D., Gee G.C. (2006). National environmental health measures for minority and low-income populations: Tracking social disparities in environmental health. Environ. Res..

[3] Fiscella K., Williams D.R. (2004). Health disparities based on socioeconomic inequities: Implications for urban health care. Acad. Med..

[4] Falkner B., Cossrow N.D. (2014). Prevalence of metabolic syndrome and obesity-associated hypertension in the racial ethnic minorities of the United States. Curr. Hypertens. Rep..

[5] Wang Y., Beydoun M.A. (2007). The obesity epidemic in the United States—Gender, age, socioeconomic, racial/ethnic, and geographic characteristics: A systematic review and meta-regression analysis. Epidemiol. Rev..

[6] Zhang Q., Wang Y., Huang E.S. (2009). Changes in racial/ethnic disparities in the prevalence of Type 2 diabetes by obesity level among US adults. Ethn. Health.

[7] Sharma S., Malarcher A.M., Giles W.H., Myers G. (2004). Racial, ethnic and socioeconomic disparities in the clustering of cardiovascular disease risk factors. Ethn. Dis..

[8] August K.J., Sorkin D.H. (2011). Racial/ethnic disparities in exercise and dietary behaviors of middle-aged and older adults. J. Gen. Intern. Med..

[9] Zavala V.A., Bracci P.M., Carethers J.M., Carvajal-Carmona L., Coggins N.B., Cruz-Correa M.R., Davis M., de Smith A.J., Dutil J., Figueiredo J.C. (2021). Cancer health disparities in racial/ethnic minorities in the United States. Br. J. Cancer.

[10] Willett W.C., Koplan J.P., Nugent R., Dusenbury C., Puska P., Gaziano T.A. (2006). Prevention of chronic disease by means of diet and lifestyle changes. Disease Control Priorities in Developing Countries.

[11] Jacques P.F., Tucker K.L. (2001). Are Dietary Patterns Useful for Understanding the Role of Diet in Chronic Disease.

[12] U.S. Department of Agriculture and U.S. Department of Health and Human Services. Dietary Guidelines for Americans, 2020–2025. https://www.dietaryguidelines.gov/sites/default/files/2020-12/Dietary_Guidelines_for_Americans_2020-2025.pdf.

[13] Richardson A.S., Arsenault J.E., Cates S.C., Muth M.K. (2015). Perceived stress, unhealthy eating behaviors, and severe obesity in low-income women. Nutr. J..

[14] Lucan S.C., Barg F.K., Karasz A., Palmer C.S., Long J.A. (2012). Concepts of healthy diet among urban, low-income, African Americans. J. Community Health.

[15] Ayala G.X., Baquero B., Klinger S. (2008). A systematic review of the relationship between acculturation and diet among Latinos in the United States: Implications for future research. J. Am. Diet. Assoc..

[16] Storey M., Anderson P. (2014). Income and race/ethnicity influence dietary fiber intake and vegetable consumption. Nutr. Res..

[17] Lee-Kwan S.H., Moore L.V., Blanck H.M., Harris D.M., Galuska D. (2017). Disparities in state-specific adult fruit and vegetable consumption—United States, 2015. MMWR. Morb. Mortal. Wkly. Rep..

[18] Breslin P.A. (2013). An evolutionary perspective on food and human taste. Curr. Biol..

[19] Gutierrez R., Fonseca E., Simon S.A. (2020). The neuroscience of sugars in taste, gut-reward, feeding circuits, and obesity. Cell. Mol. Life Sci..

[20] El-Sohemy A., Stewart L., Khataan L., Fontaine-Bisson B., Kwong P., Ozsungur S., Cornelis M.C. (2007). Nutrigenomics of taste–impact on food preferences and food production. Nutr. Oppor. Asia.

[21] Negri R., Di Feola M., Di Domenico S., Scala M.G., Artesi G., Valente S., Smarrazzo A., Turco F., Morini G., Greco L. (2012). Taste perception and food choices. J. Pediatric Gastroenterol. Nutr..

[22] Pollard J., Kirk S.L., Cade J.E. (2002). Factors affecting food choice in relation to fruit and vegetable intake: A review. Nutr. Res. Rev..

[23] Fish C.A., Brown J.R., Quandt S.A. (2015). African American and Latino low income families’ food shopping behaviors: Promoting fruit and vegetable consumption and use of alternative healthy food options. J. Immigr. Minority Health.

[24] Ko L.K., Rodriguez E., Yoon J., Ravindran R., Copeland W.K. (2016). A brief community-based nutrition education intervention combined with food baskets can increase fruit and vegetable consumption among low-income Latinos. J. Nutr. Educ. Behav..

[25] Control C.F.D. Adult Obesity Facts. https://www.cdc.gov/obesity/data/adult.html#:~:text=The%20US%20obesity%20prevalence%20was,from%204.7%25%20to%209.2%25.

[26] Bowen D.J., Barrington W.E., Beresford S.A. (2015). Identifying the effects of environmental and policy change interventions on healthy eating. Annu. Rev. Public Health.

[27] Lyn R., Aytur S., Davis T.A., Eyler A.A., Evenson K.R., Chriqui J.F., Cradock A.L., Goins K.V., Litt J., Brownson R.C. (2013). Policy, systems, and environmental approaches for obesity prevention: A framework to inform local and state action. J. Public Health Manag. Pract. JPHMP.

[28] Story M., Kaphingst K.M., Robinson-O’Brien R., Glanz K. (2008). Creating healthy food and eating environments: Policy and environmental approaches. Annu. Rev. Public Health.

[29] Mah C.L., Luongo G., Hasdell R., Taylor N.G., Lo B.K. (2019). A systematic review of the effect of retail food environment interventions on diet and health with a focus on the enabling role of public policies. Curr. Nutr. Rep..

[30] Wilkins E., Radley D., Morris M., Hobbs M., Christensen A., Marwa W.L., Morrin A., Griffiths C. (2019). A systematic review employing the GeoFERN framework to examine methods, reporting quality and associations between the retail food environment and obesity. Health Place.

[31] Cobb L.K., Appel L.J., Franco M., Jones-Smith J.C., Nur A., Anderson C.A. (2015). The relationship of the local food environment with obesity: A systematic review of methods, study quality, and results. Obesity.

[32] Janda K.M., Ranjit N., Salvo D., Nielsen A., Akhavan N., Diaz M., Lemoine P., Casnovsky J., van den Berg A. (2021). A Multi-Pronged Evaluation of a Healthy Food Access Initiative in Central Texas: Study Design, Methods, and Baseline Findings of the FRESH-Austin Evaluation Study. Int. J. Environ. Res. Public Health.

[33] US Census Bureau Quick Facts: Austin, Texas. https://www.census.gov/quickfacts/fact/table/austincitytexas/LND110210.

[34] City of Austin Office of Sustainability. Food Access in Austin. https://www.arcgis.com/apps/Cascade/index.html?appid=ddf4807ce0ad4304a8fef38f769ab14b.

[35] U.S. Bureau of the Census (2018). 2013–2017 American Community Survey 5-Year Estimates.

[36] Texas Hunger Initiative and Texas Food Bank Initiative Hunger in Travis County. http://www.austintexas.gov/edims/document.cfm?id=157974.

[37] United Way for Greater Austin (2019). 2019 Community Needs & Trends Report.

[38] City of Austin. Fresh for Less. https://www.austintexas.gov/department/fresh-less.

[39] The Food Trust (2014). Healthy Corner Store Initiative—Overview. http://thefoodtrust.org/uploads/media_items/healthy-corner-store-overview.original.pdf.

[40] Giabbanelli P.J., Crutzen R. (2017). Using agent-based models to develop public policy about food behaviours: Future directions and recommendations. Comput. Math. Methods Med..

[41] Maglio P.P., Mabry P.L. (2011). Agent-based models and systems science approaches to public health. Am. J. Prev. Med..

[42] Badham J., Chattoe-Brown E., Gilbert N., Chalabi Z., Kee F., Hunter R.F. (2018). Developing agent-based models of complex health behaviour. Health Place.

[43] Salvo D., Garcia L., Reis R.S., Stankov I., Goel R., Schipperijn J., Hallal P.C., Ding D., Pratt M. (2021). Physical activity promotion and the United Nations Sustainable Development Goals: Building synergies to maximize impact. J. Phys. Act. Health.

[44] Reis R.S., Salvo D., Ogilvie D., Lambert E.V., Goenka S., Brownson R.C., Committee L.P.A.S.E. (2016). Scaling up physical activity interventions worldwide: Stepping up to larger and smarter approaches to get people moving. Lancet.

[45] U.S. Bureau of Economic Analysis (2017). Gross Domestic Product by Metropolitan Area. https://www.bea.gov/news/2018/gross-domestic-product-metropolitan-area-2017.

[46] Sun Z., Lorscheid I., Millington J.D., Lauf S., Magliocca N.R., Groeneveld J., Balbi S., Nolzen H., Müller B., Schulze J. (2016). Simple or complicated agent-based models? A complicated issue. Environ. Model. Softw..

[47] Cocores J.A., Gold M.S. (2009). The Salted Food Addiction Hypothesis may explain overeating and the obesity epidemic. Med. Hypotheses.

[48] Filgueiras A.R., de Almeida V.B.P., Nogueira P.C.K., Domene S.M.A., da Silva C.E., Sesso R., Sawaya A.L. (2019). Exploring the consumption of ultra-processed foods and its association with food addiction in overweight children. Appetite.

[49] Krebs-Smith S.M., Pannucci T.E., Subar A.F., Kirkpatrick S.I., Lerman J.L., Tooze J.A., Wilson M.M., Reedy J. (2018). Update of the healthy eating index: HEI-2015. J. Acad. Nutr. Diet..

[50] Nielsen A., Salvo D., Ranjit N., Janda K., Zhang Y., van den Berg A. Sociodemographic factors associated with economic-, food security-and health-related concerns during COVID-19 among adults from low-income, majority-minority urban areas. Proceedings of the APHA 2021 Annual Meeting and Expo.

[51] Van den Berg A., Nielsen A., Akhavan N., Pulido C.L., Basu S., Hussaini A., Jovanovic C., Janda K., Denis L., Ranjit N. (2019). Design and evaluation of a coalition-led obesity initiative to promote healthy eating and physical activity in low-income, ethnically diverse communities: The Go! Austin/Vamos! Austin initiative. Arch. Public Health.

[52] Chiva M. (1997). Cultural aspects of meals and meal frequency. Br. J. Nutr..

[53] Post R.C. (2011). A new approach to Dietary Guidelines communications: Make MyPlate, your plate. Child. Obes. (Former. Obes. Weight Manag.).

[54] Young C.R., Aquilante J.L., Solomon S., Colby L., Kawinzi M.A., Uy N., Mallya G. (2013). Improving fruit and vegetable consumption among low-income customers at farmers markets: Philly Food Bucks, Philadelphia, Pennsylvania, 2011. Prev. Chronic Dis..

[55] Polacsek M., Moran A., Thorndike A.N., Boulos R., Franckle R.L., Greene J.C., Blue D.J., Block J.P., Rimm E.B. (2018). A supermarket double-dollar incentive program increases purchases of fresh fruits and vegetables among low-income families with children: The Healthy Double Study. J. Nutr. Educ. Behav..

[56] Durward C.M., Savoie-Roskos M., Atoloye A., Isabella P., Jewkes M.D., Ralls B., Riggs K., LeBlanc H. (2019). Double Up Food Bucks participation is associated with increased fruit and vegetable consumption and food security among low-income adults. J. Nutr. Educ. Behav..

[57] Fair Food Network Double up Food Bucks: Growing Better Access to Better Food. https://fairfoodnetwork.org/projects/double-up-food-bucks/.

[58] Pitts S.B.J., Bringolf K.R., Lloyd C.L., McGuirt J.T., Lawton K.K., Morgan J. (2013). Peer reviewed: Formative evaluation for a healthy corner store initiative in pitt county, North Carolina: Engaging stakeholders for a healthy corner store initiative, part 2. Prev. Chronic Dis..

[59] Wensel C., Trude A., Poirier L., Alghamdi R., Trujillo A., Anderson Steeves E., Paige D., Gittelsohn J. (2019). B’more Healthy Corner Stores for Moms and Kids: Identifying Optimal Behavioral Economic Strategies to Increase WIC Redemptions in Small Urban Corner Stores. Int. J. Environ. Res. Public Health.

[60] Gittelsohn J., Rowan M., Gadhoke P. (2012). Interventions in small food stores to change the food environment, improve diet, and reduce risk of chronic disease. Prev. Chronic Dis..

[61] Lent M.R., Vander Veur S.S., McCoy T.A., Wojtanowski A.C., Sandoval B., Sherman S., Komaroff E., Foster G.D. (2014). A randomized controlled study of a healthy corner store initiative on the purchases of urban, low-income youth. Obesity.

[62] Blakely T., Cleghorn C., Mizdrak A., Waterlander W., Nghiem N., Swinburn B., Wilson N., Mhurchu C.N. (2020). The effect of food taxes and subsidies on population health and health costs: A modelling study. Lancet Public Health.

[63] Choi S.E., Seligman H., Basu S. (2017). Cost effectiveness of subsidizing fruit and vegetable purchases through the Supplemental Nutrition Assistance Program. Am. J. Prev. Med..

[64] Waterlander W.E., de Boer M.R., Schuit A.J., Seidell J.C., Steenhuis I.H. (2013). Price discounts significantly enhance fruit and vegetable purchases when combined with nutrition education: A randomized controlled supermarket trial. Am. J. Clin. Nutr..

[65] Toft U., Winkler L., Mikkelsen B., Bloch P., Glümer C. (2017). Discounts on fruit and vegetables combined with a space management intervention increased sales in supermarkets. Eur. J. Clin. Nutr..

[66] Rummo P.E., Noriega D., Parret A., Harding M., Hesterman O., Elbel B.E. (2019). Evaluating a USDA program that gives SNAP participants financial incentives to buy fresh produce in supermarkets. Health Aff..

[67] Widener M.J., Metcalf S.S., Bar-Yam Y. (2013). Agent-based modeling of policies to improve urban food access for low-income populations. Appl. Geogr..

[68] Salvo D., Ranjit N., Nielsen A., Akhavan N., van den Berg A. (2019). Characterizing micro-scale disparities in childhood obesity: Examining the influence of multilevel factors on 4-year changes in BMI, healthy eating, and physical activity, among a cohort of children residing in disadvantaged urban enclaves. Front. Public Health.

[69] Evans A.E., Jennings R., Smiley A.W., Medina J.L., Sharma S.V., Rutledge R., Stigler M.H., Hoelscher D.M. (2012). Introduction of farm stands in low-income communities increases fruit and vegetable among community residents. Health Place.

[70] Ranjit N., Nielsen A., Akhavan N., Denis L., Janda K., Jovanovic C., Basu S., Hussaini A., van den Berg A. (2020). Outcomes of a community-wide health intervention in a low-income, primarily Hispanic community: The Go! Austin/Vamos! Austin (GAVA) Initiative. Health Promot. Pract..

